# Real-time Forecast of Multiphase Outbreak

**DOI:** 10.3201/eid1201.050396

**Published:** 2006-01

**Authors:** Ying-Hen Hsieh, Yuan-Sen Cheng

**Affiliations:** * National Chung Hsing University, Taichung, Taiwan

**Keywords:** SARS, emerging infectious disease, Canada, Toronto, turning point, Richards model, case data, public health, research

## Abstract

We used a single equation with discrete phases to fit the daily cumulative case data from the 2003 severe acute respiratory syndrome outbreak in Toronto. This model enabled us to estimate turning points and case numbers during the 2 phases of this outbreak. The 3 estimated turning points are March 25, April 27, and May 24. The estimated case number during the first phase of the outbreak between February 23 and April 26 is 140.53 (95% confidence interval [CI] 115.88–165.17) if we use the data from February 23 to April 4; and 249 (95% CI: 246.67–251.25) at the end of the second phase on June 12 if we use the data from April 28 to June 4. The second phase can be detected by using case data just 3 days past the beginning of the phase, while the first and third turning points can be identified only ≈10 days afterwards. Our modeling procedure provides insights into ongoing outbreaks that may facilitate real-time public health responses.

## Abstract 2

The multistage Richards model provides insights into ongoing outbreaks that may be useful for real-time public health responses.

Mathematical models have been used to predict the course of epidemics, albeit with mixed results (*1*). Whether and how infectious diseases are likely to spread (*2**–**4*) are affected by stochastic events (*5*). Once outbreaks have begun, knowing their potential severity helps public health authorities respond immediately and effectively. Much relevant information is contained in the answers to 2 questions: 1) Is the current outbreak getting better or worse? 2) How many people will be infected before the outbreak ends? Attempts to answer these questions in the early stages of an epidemic can be futile and at times misleading (*6*); nonetheless, we can address them with an appropriate mathematical model once sufficient time has elapsed (*7*). Moreover, answers can be accurate if no stochastic event occurs that could substantially alter the course of outbreaks.

We use a variation of the single-equation Richards model (*8*) to answer these key questions. Unlike models with several compartments commonly used to predict the spread of disease, the Richards model considers only the cumulative infective population size with saturation in growth as the outbreak progresses, caused by decreases in recruitment because of attempts to avoid contacts (e.g., wearing facemask) and implementation of control measures.

The basic premise of the Richards model is that the daily incidence curve consists of a single peak of high incidence, resulting in an S-shaped epidemic curve and a single turning point of the outbreak. These turning points, defined as times at which the rate of accumulation changes from increasing to decreasing or vice versa, can be easily located by finding the inflection point of the epidemic curve, the moment at which the trajectory begins to decline. This quantity has obvious epidemiologic importance, indicating either the beginning (i.e., moment of acceleration after deceleration) or end (i.e., moment of deceleration after acceleration) of a phase. The Richards model fits the single-phase severe acute respiratory syndrome (SARS) outbreaks in Hong Kong and Taiwan (*7**,**9*) well. However, in the case of the Toronto outbreak, the second wave of nosocomial infections in May caused the epidemic curve to deviate from the standard S shape. We propose an improvised version of the Richards model that fits the epidemic in Toronto and, subsequently, provide a simple procedure for real-time forecasts of outbreaks with secondary and tertiary waves.

## Methods

The Richards model is logistic and is described by a single differential equation. The equation is given below, where *I(t)* is the cumulative number of infected cases at time *t* in days:(1)_
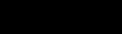
_The solution is:


(2)_
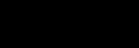
_


During initial stages of the outbreak, when *I*(*t*) is small compared to *K*, the growth rate *r* is approximated by _
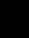
_, or roughly the number of cases on day *t* over the cumulative case number through that day. We can show mathematically that *t_i_* is the only inflection point (or turning point denoting deceleration after acceleration) of the epidemic curve obtained from this model. Moreover, *t_m_* = *t_i_*+ (*lna*)/*r* is equal to the inflection point *t_i_* when *a* = 1 and approximates *t_i_* when *a* is close to 1

**Figure 1:**
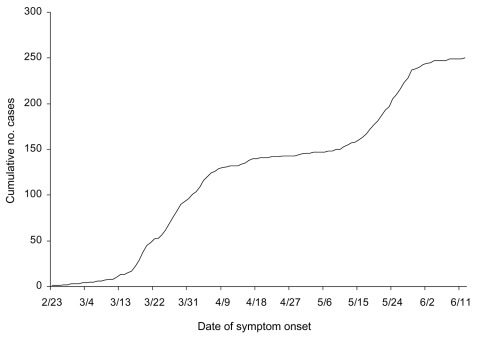
Cumulative severe acute respiratory syndrome cases by onset of symptoms for 250 cases in Canada, February 23– June 12, 2003 (1 case had unknown onset). All except 1 of the 250 cases were in Toronto area (http://www.phac-aspc.gc.ca/sars-sras/pdf-ec/ec_20030808.pdf).

The model parameters are as follows: *K* is the carrying capacity or total case number, *r* is the per capita growth rate of the infected population, and *a* is the exponent of deviation from the standard logistic curve. Because the Richards model typically exhibits a single S-shaped curve, it is not suitable for the SARS epidemic in Canada illustrated in Figure 1.

**Figure 2:**
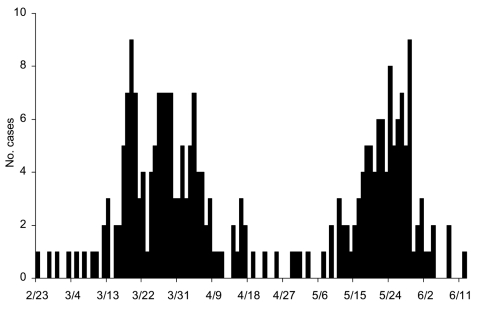
Severe acute respiratory syndrome incidence curve for Toronto area, February 23–June 12, 2003.

To rectify this situation, we proposed a multistage Richards model, 1 stage for each of the S-shaped segments resulting from multiple waves of infection during this outbreak. Stages are distinguished by turning points (or inflection points), denoting acceleration after deceleration at the end of each S-shaped segment, the local minima of the corresponding incidence curves. For an *n*-phase epidemic outbreak, *n* – 1 local minima separate the *n* phases. For illustration, the incidence curve for Toronto given in Figure 2 contains 2 peaks (local maximum or turning point of the first type) and 1 valley (local minimum or turning point of second type). The multistage Richards model procedure requires 5 steps. First, fit the Richards model to cumulative cases on successive days by using a standard least-square routine. For single-phase outbreaks, parameter estimates (*a*, *r*, *t_i_*, *K*) will converge as the trajectory approaches carrying capacity *K*, as demonstrated in the Taiwan and Hong Kong SARS outbreaks (*7**,**9*). Second, if estimated parameters remain convergent until no more new cases are detected, the outbreak has only 1 phase. However, if the estimates begin to diverge from heretofore fixed values, one knows that a turning point denoting the start of a second phase has occurred. Third, locate the turning point, *t_min_*, separating 2 S-shaped phases of the epidemic as the local minimum of the incidence curve (Figure 2). This is the curve for S´(*t*) given in the equation (*1*). Fourth, fit the Richards model to the cumulative case curve again, but starting from *t_min_* + 1, the day after the start of second phase. The estimated parameters (*a*, *r*, *t_i_*, *K*) will again converge as the curve approaches the carrying capacity *K* for the second phase. Finally, repeat steps 2–4 in the event more phases occur until the outbreak ends.

By considering successive S-shaped segments of the epidemic curve separately, one can estimate the maximum case number, *K*, and locate the turning points, thus providing an estimate for the cumulative number of cases during each phase.

## Results

For the phase starting February 23, we estimate parameters from data ending on various dates in Table 1. We could obtain estimates for every consecutive day after recognizing the outbreak, but we only give results for every 10 days for brevity, with the first ending on March 25. The best fitting Richards model, ending on April 26 and 28, yields the parameter values given in bold letters. The estimated value for the turning point *t_i_* during this phase is computed from the estimates for *r*, *a*, and *t_m_* by using equation (*2*). As the initial time *t* = 0 is February 23 and symptom onset occurs ≈5 days after infection (*10*), *t_i_* = 30.43 gives the first inflection point around March 25 or first turning point (from acceleration to deceleration) for disease transmission in the Toronto area ≈5 days before March 20.

**Table 1:** Estimates of parameters for Richards model using cumulative case data of selected time periods in phase 1 of 2003 Toronto area SARS outbreak starting from February 23 with 95% confidence interval for the maximum case number K*

End date	Growth rate	Exponent of deviation	Turning point	Maximum case no.
Mar 25	0.859	4.835	25.09	60.10 (54.71–65.49)
Apr 4	0.146	0.689	30.06	140.53 (115.88–165.17)
Apr 14	0.152	0.773	30.50	142.78 (137.34–148.22)
Apr 24	0.147	0.718	30.45	143.99 (141.76–146.21)
Apr 26	0.146	0.710	30.43	144.14 (142.19–146.09)
Apr 28	0.146	0.709	30.43	144.14 (142.42–145.86)
Apr 30	0.144	0.693	30.40	144.41 (142.85–145.96)
May 2	0.142	0.664	30.35	144.84 (143.40–146.29)

*SARS, severe acute respiratory syndrome.

**Figure 3:**
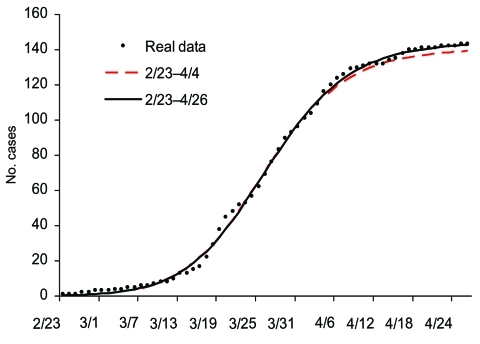
pidemic curves for the first phase of severe acute respiratory syndrome outbreak in Toronto area using multistage Richards model and cases, February 23– April 26, 2003.

The number of cases during the phase ending on April 26 is 144, well approximated by our carrying capacity, *K* = 144.14 (95% confidence interval [CI] 142.19–146.09). Moreover, the results in Table 1 show that, using data from February 23 to April 4, or 10 days after the turning point of this phase, model fitting gives an estimate of *K* = 140.53 (95% CI 115.88–165.17). That is, given case data at the time of the outbreak, we could estimate the cumulative case number in the first phase accurately (Figure 3) 10 days after the turning point on March 25 and 22 days before the end of the first phase. This estimate also is the cumulative case number assuming no subsequent waves of infection.

Unfortunately, this was only the first wave in this outbreak, as indicated by estimates starting to diverge again after April 30. The last 2 rows of Table 1 suggest that the second turning point, the start of a second phase of this outbreak, occurred by April 30. Consequently, we go to step 3 in our procedure.

Here we use the incidence data starting on April 18 and continuing past April 30 to obtain a least-squares estimate of the minimum point *t_min_* of the incidence curve. This choice of period ensures the minimum is contained in the time interval. Given that *t = 0* is April 18, the least-squared estimate of the local minimum converges after May 18 and is *t_min_* = 9.11 (95% CI 8.95–9.27) as shown in Table 2, along with previous estimates given every other day. This finding pinpoints the second turning point of the Toronto outbreak at April 27. Hence, April 27 separates the 2 S-shaped curves spanning the respective time periods February 23 to April 26 and April 28 to June 12, the end of the outbreak.

**Table 2:** Estimates of *t_min_* using incidence curve starting on April 18

End date	Turning point	95% CI*
Apr 30	5.08	4.92–5.24
May 2	5.54	5.38–5.70
May 4	4.83	4.67–4.99
May 6	7.20	7.04–7.36
May 8	8.18	8.02–8.34
May 10	6.50	6.34–6.66
May 12	8.18	8.02–8.34
May 14	7.65	7.49–7.81
May 16	8.08	7.92–8.24
May 18	9.11	8.95–9.27
May 20	9.11	8.95–9.27

*CI, confidence interval.

Again, as the data used in this article are given by onset date, which occurred after ≈5 days of incubation (*10*), April 22 is the actual second turning point that foretold the second wave of infections in Toronto. The index patient for the second phase had onset of respiratory symptoms, fever, and diarrhea on April 19 (*11*), 3 days before the turning point pinpointed by this procedure. Our result also corroborates the assessment of Health Canada, which pinpointed April 21 as the start of second phase of the outbreak in Toronto (Figure 1 in [11]).

**Figure 4:**
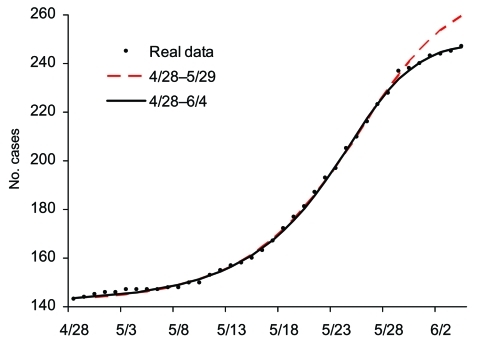
Epidemic curves for the second phase of severe acute respiratory syndrome outbreak in Toronto area using multistage Richards model and cases, April 28–June 4, 2003.

Starting with the second phase of the outbreak on April 28, we again fit the cumulative case data from April 28 to the Richards model. As the case number on April 28 is 144, we use a transformation of *S*(*t*) = *S_real_*(*t*)–143, where *S_real_*(*t*) is the actual data at time *t*, so the initial data on April 28 used here is *S*(0) = 1. We again fit the model to the cumulative data ending on various dates past May 25; the results are given in Table 3 and Figure 4. The estimates start to converge after June 4, in the last 2 rows of Table 3 in bold, yielding an estimate for *K* of 248.96 (95% CI 246.67–251.25). Once again, the actual case number of 249 for the Toronto area outbreak (and 250 for Canada) is well approximated by our estimate of *K*. The estimated turning point *t_i_* = 26.36 pinpoints May 24, or a turning point for SARS infections 5 days earlier on May 19. This finding further corroborates Health Canada's assertion that, among the 79 cases that resulted from exposure at the hospital where the index patient of the second phase stayed, 78 had exposures that occurred before May 23 (*11*). Note also that this estimate is obtained by using data that end just 11 days after the turning point on May 24, giving an accurate prediction of the actual cumulative case number (Figure 4).

**Table 3:** Estimates of parameters for Richards model using cumulative case data of selected time periods in phase 2 of 2003 Toronto area SARS outbreak starting from April 28 with 95% confidence interval for the maximum case number K*

End date	Growth rate	Exponent of deviation	Turning point	Maximum case no.
May 25	0.557	3.866	24.59	223.37 (199.67–247.07)
May 27	0.350	2.393	25.84	244.36 (220.53–268.18)
May 29	0.236	1.554	27.36	271.28 (240.94–301.62)
May 31	0.321	2.202	26.43	252.53 (244.32–260.74)
Jun 2	0.352	2.448	26.36	249.51 (245.70–253.33)
Jun 4	0.359	2.508	26.36	248.96 (246.67–251.25)
Jun 6	0.367	2.576	26.37	248.52 (246.98–250.07)

*SARS, severe acute respiratory syndrome.

## Discussion

We show that the first turning point on March 25 could be detected 10 days after it occurred on April 4 (row 2 in Table 1). The second turning point on April 27, indicating that the epidemic escalated again, could be detected 5 days after it occurred by May 2 (last row in Table 1 shows the estimate for *t_i_* diverging). And the third turning point on May 24 could be detected 7 days after it occurred on May 31 (row 4 in Table 3).

**Figure 5:**
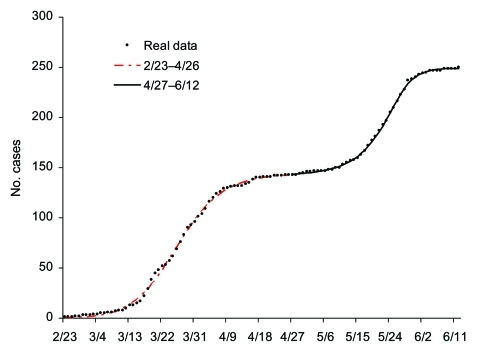
Epidemic curve for Toronto area severe acute respiratory syndrome outbreak of February 23–June 12, 2003, using multistage Richards model. Turning points are March 25, April 27, and May 24.

Our procedure fits the data well (Figure 5), allowing us to study retrospectively the significance of various events occurring at different times. Through this procedure, we can pinpoint retrospectively the 3 key turning points for the spread of disease during the 2-phase outbreak in Toronto area. The first turning point for the spread of SARS occurred on March 20 when the first wave of infections leveled off. April 22 was the second turning point, at which time persons infected by the undetected index patient for the second wave began to experience symptoms. Our findings also concur with the World Health Organization action that lifted a travel advisory issued on April 22 that limited travel to Toronto. In retrospect, the Toronto outbreak would have ended with the first wave, if not for the single undetected case and subsequent infections that occurred before April 22. Furthermore, our results also corroborate the assessment of Health Canada, which pinpointed April 21 as the start of the second phase of the outbreak in Toronto area. The third and final turning point for the infections occurred on May 19, when the spread of disease finally leveled off.

Given incidence by onset date during the outbreak, one can use our procedure to forecast the eventual severity of current phases of the outbreak by estimating the carrying capacity, *K*. However, accuracy depends on having the incidence data for some time past the inflection point (*7*) and no new waves of infection in the future. Both points can be aptly illustrated by the Toronto outbreak. By using data from 2/23–4/14, we can predict the 95% CI of cases in the first phase of this outbreak at 137.34–148.22, 10 days before the phase ended. Incidence data 20 days after the inflection point of the first phase (March 25) would have enabled us to project the severity of the epidemic, had there not been a second wave of infection. By performing daily fits with updated case data, one could determine if parameters were converging to reliable values for the current phase of the outbreak. Similarly, for phase 2 of the Toronto outbreak, 11 days after the final inflection point (May 24), the data from April 28 to June 4 give a good estimated 95% CI of the cumulative cases of 246.67–251.25, 8 days before onset of the last case.

These results can also be used to compute the basic reproduction number, *R_0_*, for the Toronto outbreak. From Table 1, *r* = 0.146 for the first phase. To compare with results (*9*), we also assume the duration of infectiousness *T* to be 8.4 days, as estimated from the time from onset of symptoms in the index patient to onset of symptoms in a secondary case-patient in Singapore (*12*) and obtain *R_0_* = *exp*[*rT*] = 3.41. The estimated *r* = 0.136 for Taiwan outbreak in (*7*) yields *R_0_* = 3.08. Note that, because of the shift in the cumulative number used for the model fit of the second phase, the resulting value for *r* cannot be used in this simple calculation. A list of basic reproduction numbers for SARS in affected areas computed in literature by using Richards model and *T* = 8.4 is given in Table 4 for comparison. The larger basic reproduction numbers for Toronto (phase 1) and Taiwan, as compared with Hong Kong and Singapore, may be attributable to the relatively high percentage of nosocomial infections (*13**,**14*).

**Table 4:** Comparison of basic reproduction numbers (R0) for SARS in some affected areas in literature computed by using Richards model and T = 8.4*

Affected area	Reference	Growth rate	*R*_0_
Singapore	*9*	0.12	2.7
Hong Kong	*9*	0.09	2.1
Taiwan	*7*	0.136	3.08
Toronto (phase I)	This article	0.146	3.41

*SARS, severe acute respiratory syndrome.

The easily implemented procedure described can be extended to analysis of turning points and severity of multiphase epidemics while ongoing. During an outbreak such as SARS, to which available data were limited and uncertain, a simple model that requires only the most basic and perhaps only easily obtainable data under these circumstances offers our best chance to a practical solution to the understanding, prediction, and timely control of the outbreak. However, one must understand that mathematical models do not provide accurate numerical predictions and can be used to forecast only in fairly gross terms (*15*). The accuracy of predictions depends heavily also on the assumption that no stochastic events occur in the remaining days that could significantly alter the course of the current phase of an outbreak.

Detecting the occurrence of a second turning point or start of a second phase, as outlined in Step 2 of our procedure, is especially useful as it allows us to recognize early that an epidemic is worsening, in our case on April 30 only 3 days after the turning point on April 27 (Table 1). Though predicated on the availability and accuracy of case onset data, this procedure could be a valuable tool to public health policymakers for responding to future disease outbreaks with multiple turning points.
